# Social Representations of nurses on the approach to children and adolescents who are victims of violence*

**DOI:** 10.1590/1518-8345.5414.3509

**Published:** 2021-11-19

**Authors:** Emanuella de Castro Marcolino, Francisco de Sales Clementino, Rafaella Queiroga Souto, Renata Clemente dos Santos, Francisco Arnoldo Nunes de Miranda

**Affiliations:** 1 Centro Universitário UNIFACISA, Departamento de Enfermagem e Medicina, Campina Grande, PB, Brazil.; 2 Universidade Federal de Campina Grande, Departamento de Enfermagem, Campina Grande, PB, Brazil.; 3 Universidade Federal da Paraíba, João Pessoa, PB, Brazil.; 4 Universidade Federal do Rio Grande do Norte, Natal, RN, Brazil.

**Keywords:** Nurses, Child Abuse, Child, Adolescent, Health Services, Forensic Nursing, Enfermeras y Enfermeros, Maltrato Infantil, Niño, Adolescente, Servicios de Salud, Enfermería Forense, Enfermeiras e Enfermeiros, Maus-Tratos Infantis, Criança, Adolescente, Serviços de Saúde, Enfermagem Forense

## Abstract

**Objective:**

to analyze social representations from the perspective of the structural aspect about the nurses’ approach to children and adolescents who are victims of violence, comparing primary, secondary and tertiary health care services.

**Method:**

an analytical research study with a qualitative approach under the methodological theoretical framework of the Theory of Social Representations from the Central Core Theory. A total of 76 nurses participated in the study: 30 from primary care, 16 from secondary care and 30 from tertiary care. A semi-structured interview was applied using a pre-defined script and similarity analysis using the *Interface of R pour les Analyses Multidimensionnelles de Textes et de Questionnaires* software.

**Results:**

structurally, the maximum tree revealed the central core in the upper right quadrant, the first peripheral zone in the upper left quadrant; the second peripheral zone in the lower left quadrant; and the silent zone in the lower right quadrant. The ten branches of the maximum tree emerged from the following terms: *hit, leave, approach (n), receive, approach (v), remember, tell, spend, pass, caution, mom*.

**Conclusion:**

the social representations on the nurses’ approach in primary, secondary and tertiary care health services evidenced common points as for the lack of notification, transfer of responsibilities, weakness in identifying situations of violence and the need for training.

## Abstract (es)

**Objetivo:**

analizar las representaciones sociales desde la perspectiva de la vertiente estructural sobre el abordaje del enfermero a los niños y adolescentes víctimas de violencia, comparando los servicios de atención primaria, secundaria y terciaria.

**Método:**

investigación analítica con enfoque cualitativo basada en el marco teórico metodológico de la Teoría de las Representaciones Sociales a partir de la Teoría del Núcleo Central. En el estudio participaron 76 enfermeros en total, 30 de atención primaria, 16 de atención secundaria y 30 de atención terciaria. Se aplicó una entrevista semiestructurada utilizando un cuestionario predefinido y el análisis de similitud a través del software *Interface de R pour les Analyses Multidimensionnelles de Textes et de Questionnaires*.

**Resultados:**

estructuralmente, el árbol máximo reveló el núcleo central en el cuadrante superior derecho, la primera zona periférica en el cuadrante superior izquierdo; la segunda zona periférica en el cuadrante inferior izquierdo y en el cuadrante inferior derecho la zona muda. Las diez ramas del árbol máximo surgieron de los términos: *golpear, dejar, abordaje, recibir, abordar, recordar, contar, pasar, cuidado, madre*.

**Conclusión:**

las representaciones sociales del abordaje de los enfermeros en los servicios de atención primaria, secundaria y terciaria de la salud demostraron que tenían puntos en común en cuanto a la falta de notificación, transferencia de responsabilidades, deficiencia en la identificación de situaciones de violencia y necesidad de capacitación.

## Abstract (pt)

**Objetivo:**

analisar as representações sociais na perspectiva da vertente estrutural sobre a abordagem do enfermeiro às crianças e adolescentes vítimas de violência, comparando-se serviços de saúde de atenção primária, secundária e terciária.

**Método:**

pesquisa analítica com abordagem qualitativa sob o aporte teórico metodológico da Teoria das Representações Sociais a partir da Teoria do Núcleo Central. Participaram do estudo 76 enfermeiros, sendo 30 da atenção primária, 16 da atenção secundária e 30 da atenção terciária. Aplicou-se entrevista semiestruturada por meio de roteiro pré-definido e análise de similitude a partir do *software Interface de R pour les Analyses Multidimensionnelles de Textes et de Questionnaires*.

**Resultados:**

estruturalmente, a árvore máxima revelou o núcleo central no quadrante superior direito, a primeira zona periférica no quadrante superior esquerdo; a segunda zona periférica no quadrante inferior esquerdo e no quadrante inferior direito a zona muda. As dez ramificações da árvore máxima emergiram a partir dos termos: *bater, deixar, abordagem, receber, abordar, lembrar, contar, passar, cuidado, mãe*.

**Conclusão:**

as representações sociais da abordagem dos enfermeiros nos serviços de saúde de atenção primária, secundária e terciária evidenciaram pontos em comum quanto a déficit de notificação, transferência de responsabilidades, fragilidade na identificação das situações de violência e necessidade de capacitação.

## Introduction

Maltreatment of children and young individuals represent an international, national and regional reality affecting children and adolescents from different cultural, ethnic and social contexts^(1)^. This phenomenon is shown as a demand for health services around the world. Currently, there is an urgent need for approaches with an integral and contextual view for these victims, requiring nurses’ skills and expertise to manage these situations of violence^(2)^.

According to the World Health Organization (WHO), violence against children and adolescents consists of all forms of physical, emotional and sexual harm, abandonment, exploitation or neglect carried out by oppressive power relations affecting children’s and adolescents’ development and dignity^(1)^; with the main forms of abuse being as follows: negligence, and physical, psychological and sexual abuse^(3)^.

In this scenario of child-youth vulnerability, eleven of the 18 Sustainable Development Goals (SDGs) and 19 of the 53 health-related SDG indicators are related to children’s and adolescents’ health; the protection of this vulnerable group being a global commitment of the international agencies; however, it is estimated that, globally, every year one out of two children aged from 2 to 17 years old suffer some form of violence; one third of the adolescents aged between 11 and 15 years old are bullied by their peers in schools and 120 million girls under the age of 20 have suffered sexual violence^(4)^.

In North America, the lifetime prevalence of sexual abuse is 20% for girls; and psychological/emotional violence is 28%; while in South America, the indicators show a high prevalence of neglect with numbers of 55% for girls and 57% for boys^(4)^. In the Brazilian scenario, interpersonal violence is the second or third leading cause of death among children and adolescents, depending on the region^(5)^. In the national scenario, in 2019, DISK 100 data indicated the occurrence of 17,000 reports of sexual violence against children and adolescents, with 73% of the cases taking place in the victim’s home and 46% of the victims being female adolescents (12-18 years old)^(6)^.

Violence against children and adolescents is a public health problem of high priority; according to the WHO. Among the seven strategies to combat violence against children and adolescents in the world, access to good quality health, protection and justice services stand out^(7)^. Thus, nurses have proved to be key elements in the prevention process, early identification and assistance of child-youth abuse^(8)^; for this, it becomes necessary that such professionals raise suspicions regarding such cases in the various health services^(9)^.

However, the nurses’ role in coping with violence situations is still permeated by several challenges involving professional qualification during academic training and/or permanent education at work, as well as difficulties in reporting the cases, need for protocols and care routines^(10)^.

A number of studies^(11-15)^ point out relevant factors that permeate the intervention by nurses in cases of violence in the pediatric and adolescent population, such as the reason for the silence established in the family, the fear coming from the professionals for having doubts about tangible problem solving, definition of care protocols and flows, professional training, lack of resoluteness by the child protection agencies and lack of institutional and governmental support to deal with these families^(16)^.

From this perspective, apprehension of the convergences and divergences in the nurses’ approach to children and adolescents who are victims of violence raises the need to understand the social representations of the nurses’ performance in different health care settings. The nurses’ role in primary health care is marked by the bond with the communities, which, on the one hand, favors the identification of situations of violence and, on the other hand, causes fears and insecurities in the face of the cases^(17)^; in the hospital service, nurses are in an emergency context with difficulties in correctly identifying and reporting abuse situations with children and adolescents^(18)^.

The theory of social representations is based on the analysis of socially elaborated and shared knowledge in specific processes of social interaction, which contributes to the formation of a common reality in a given social group, showing what is consciously shared with other members of the social group^(19)^.

However, there are still no research studies that reach the nurses’ social representations^(20)^ about their professional performance in the face of child abuse in order to qualitatively understand the elements that permeate the nurses’ approach to these victims of violence considering the Brazilian organizational structure of health by levels of care and their specificities.

Based on this panorama of deficits in qualitative studies on nurses’ management of child abuse, associated with the evident need for qualification in coping with these situations in the health services, scientific deepening of the relationships that permeate this professional practice is justified.

Given the above, the following question emerged: Which are the social representations that permeate the nurses’ approach to children and adolescents who are victims of violence in health care settings? To answer the question in terms of social representations, the structural aspect was chosen as one of the apprehensions of social representations, under the Central Core Theory (CCT)^(21)^, considered as a central element of the nurses’ performance in different health services in the care of victimized children and adolescents.

The objective was to analyze the social representations from the perspective of the structural aspect of the nurse’s approach to children and adolescents who are victims of violence, comparing primary, secondary and tertiary health care services.

## Method

### Type of study

An analytical research study with a qualitative approach was carried out under the methodological theoretical framework of the Theory of Social Representations (TSR)^(22)^ based on the Central Core Theory^(22)^. The choice of such theory is justified in order to achieve translation of the meanings and values intrinsic to the nurses’ work when approaching children and adolescents in situations of violence, as historically determined beings, immersed in a particular society and culture, with an emphasis on the Central Core in order to recognize the central essence of the nurses’ approach at different health care levels.

Elaboration of the manuscript followed the COREQ (Consolidated Criteria for Reporting Qualitative Research) recommendations, meeting the scientific requirements for a qualitative study.

### Research scenario

The research was designed in a municipality from the inland of northeastern Brazil, a reference for health care in the surrounding municipalities of the state of Paraíba. In order to maximize the number of nurses working at the three levels of care for children and adolescents who are victims of violence, Family Health teams (FHts) were selected to cover primary care; a specialized hospital, for pediatric care representing secondary care; and a referral hospital, for trauma and violence at a regional level, in tertiary care.

At the time of data collection, there were 107 FHts in the municipality, distributed in 84 Basic Health Units (BHUs) and six Health Districts. The specialized hospital for pediatric care, which is characteristic of secondary care offering outpatient and specialized services, without high-complexity demand, stands out as the only pediatric hospital service in the municipality, with 25 nurses working in the reception, emergency and nursing sectors.

The tertiary level was represented by a reference hospital for trauma and violence at the regional level, which has the largest number of appointments related to serious situations of violence involving children and adolescents. There were 62 nurses distributed among the sectors: reception, red room, pediatric observation, pediatric ward and pediatric ICU.

### Study participants and selection criteria

A total of 76 nurses selected by convenience participated in the study. The number of participants was determined by theoretical saturation^(23)^ for the groups of nurses at the primary and tertiary levels and by exhaustion criteria for the professionals at the secondary level.

In this research, theoretical saturation was evidenced from the repetition of speeches and identical patterns on the way to approach the victims regarding reception and identification of the victim, case management in the multi-professional team, clinical evaluation and Nursing consultation of children and adolescents who are victims of violence; based on the recognition of this aspect of recurrence of verbalization of the facts, data collection was suspended. Exhaustion was characterized by the inclusion of all eligible individuals as study participants.

**Figure 1:**
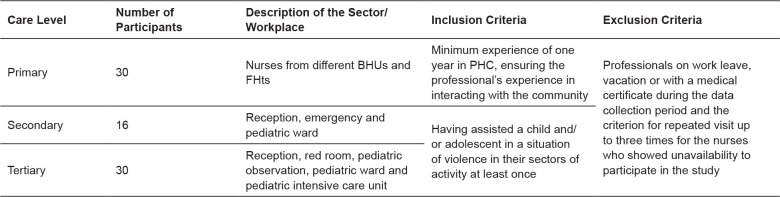
Characterization of the number of participants, description of the sector/workplace and selection criteria according to the care level. Campina Grande, Paraíba, Brazil, 2021

Figure 1 below synthesizes the number of participants included in each service level, the description of the sectors/places of operation and the selection criteria.

There were no refusals among the participants at the primary level, with one refusal in the secondary service and eight at the tertiary level associated with the dynamics of an intense work routine.

### Procedures and data collection instruments

Data collection took place between January and June 2018, performed in its entirety and exclusively by the researcher in charge in order to minimize biases by multiple collectors; the researcher had no institutional, labor or personal relationship with the institutions and/or nurses working in the health services researched.

Initially, the face-to-face approach of the tertiary care nurses was performed, according to the professional schedule, in the five sectors selected in the hospital service; subsequently, the nurses of the specialized hospital for the care of children and adolescents were approached following the same procedures; and, finally, the primary care nurses; during the approaches, the study objective and the link of the research to the graduate program were explained.

Two instruments were applied: an individual form to characterize the study participants and a semi-structured interview script used as a guide in conducting the interviews. The semi-structured interview script was based on guiding questions with a focus on the nurses’ approach to children and adolescents in situations of violence in different health services, such as: How are the actions aimed at children and adolescents in situations of violence carried out in the health service where you work?

The interviews were fully recorded by signing the voice recording form for later full transcription, lasting a mean of 20 to 30 minutes. The participating nurses were coded in the speech excerpts by means of “enf.” (“*enfermeiro*” in Portuguese) and an Arabic numeral referring to the order in which the interview was conducted; health care level and sector of activity, respectively, with “level 01” for primary care and “ds” (“*distrito de saúde*” in Portuguese) 01 to 06 referring to the health districts; “level 02” for secondary care and sectors 01 - reception, 02 - emergency, 03 - pediatric ward; and “level 03” for tertiary care and sectors 01 - reception, 02 - red room, 03 - pediatric observation, 04 - pediatric ward, 05 - pediatric ICU.

### Data treatment and analysis

The data were processed using the IRAMuTeQ (*Interface by R pour les Analyses Multidimensionnelles de Textes et de Questionnaires*) software^(24)^, which enabled production of the maximum tree (similarity analysis).

Data organization for analysis in the software occurred, initially, by the construction of the textual *corpus* according to procedures defined by IRAMuTeQ, namely: organization of the textual content in the Free Office software; definition of the command line for each interview using asterisk markers (e.g.: **** *enf_01 *ds_03 *posgrad_02 *capacit_01); construction of the content in a monothematic way with removal of the questions and paragraphs, standardization of acronyms or words composed by the underline symbol, inclusion of numbers in the form of an algorithm and exclusion of symbols such as quotation marks, apostrophes, hyphens, dollar signs, percentages, ellipses, and asterisks in the textual corpus; such organization resulted in a corpus of 186 pages.

For data analysis, the Theory of Social Representations was adopted as the methodological theoretical grounds, with emphasis on the Central Core Theory, which was considered a complementary approach to the first, considered, among others, as the structural aspect^(21)^. Based on the premise that every social representation is organized around a central core associated with other complementary structural instances, the central core has the organizing function as a unifying and stabilizing element of the representation^(25)^.

Thus, the similarity analysis was performed as a technique for surveying the central core, which is considered the main technique for detecting the degree of connectivity between the elements of a representation and, consequently, defining the central core. Similarity analysis is one of the main analysis techniques to achieve the Social Representations.

The similarity analysis, via IRAMuTeQ, was based on the relationship between the number of co-occurrences and the number of subjects involved, establishing connections between these elements based on the graph theory; the graphic representation of the connections produces the maximum tree. The parameters for the construction of the maximum tree included the co-occurrence index and the descriptive variable highlighted in the graphic representation, which is the health care level^(26)^. In the maximum tree, code I represents the terms associated with PHC nurses; code II is the secondary care service and code III is the tertiary care service in each analytical axis; those terms without coding were not associated with any specific health care level.

The maximum tree represents the organization of the nurses’ social representations when approaching victims of violence from a central core and in branches, these latter structured by the specificities of the health care level (descriptive variable); thus, the analysis provided translation of the senses and meanings that support the nurses’ social representations, showing the common and peripheral aspects of such representations in accordance with the level of care in the health service: primary, secondary and tertiary care.

With the textual corpus organized, a similarity analysis was performed to obtain the maximum tree. Subsequently, the excerpts of relevant speeches that agreed with the organization of the maximum tree regarding the approximations and distances of the nurses’ social representations were selected.

Given the structuring of the Social Representations, provided by the similarity analysis associated with text segments from the nurses’ speeches, Bardin’s analysis of co-occurrences was constituted^(27)^ in order to consolidate the common and divergent elements in the nurse’s approach to children and adolescents who are victims of violence expressed in a summary figure. The co-occurrence analysis obeys the following approach: choice of the registration units; choice of the context units; coding; calculation of co-occurrences; representation and interpretation of the data.

The analysis and intellectual structuring of the data were carried out by the main researcher in a joint effort with the research supervisor, who was an expert in the use of the Theory of Social Representations, which ensured reliability of the scope of the Social Representations evoked by the analysis outlined with the aid of the software.

### Ethical aspects

The research followed the ethical parameters of the National Health Council, being authorized by the Ethics Committee of the Federal University of Rio Grande do Norte with approval number 2,456,493.

## Results

Among the three levels of complexity, female nurses aged between 30 and 40 years old were predominant. Most of the professionals in the first and third care level had more than 10 years since graduation, while those in the specialized service had between 5 and 9 years since having finished their studies. As for the specific training to approach children and adolescents in situations of violence, most of the professionals at the three levels stated that they had not undergone any training.

The analysis of the nurses’ approach to situations of violence in children and adolescents showed similarities and distances in the management of cases among nurses in primary, secondary and tertiary care health services, as a result of the performance context, the training and the training process.

**Figure 2:**
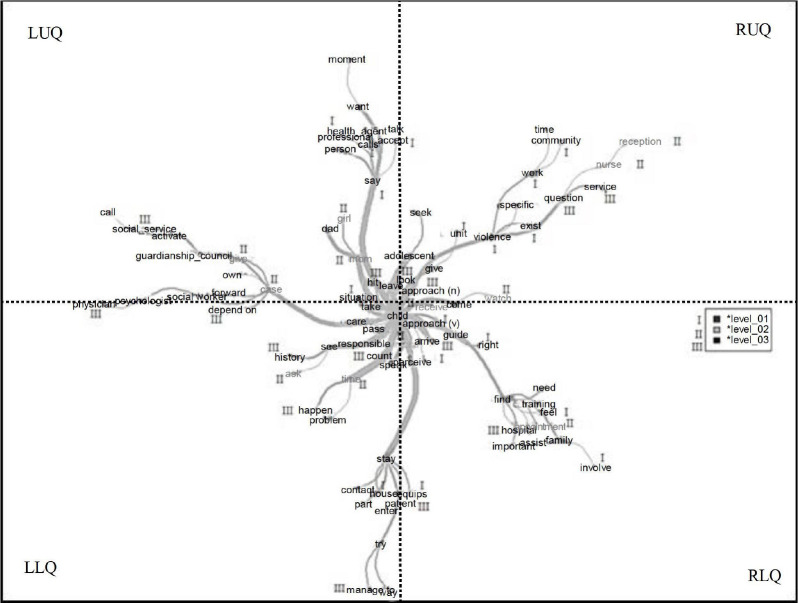
Similarity analysis of the nurses working at the three levels of complexity on the approach to children and adolescents in situations of violence. Campina Grande, Paraíba, Brazil, 2021 * I - Primary care;^†^II - Secondary care;^‡^III - Tertiary care

Figure 2 represents the maximum tree produced by the similarity analysis that reveals the central core of the nurses’ social representation regarding the care of children and adolescents in situations of violence. The focus of the approach to children is perceived as a common element among nurses at the three health care levels, a representational centrality based on the nucleation of the nurses’ statements.

The maximum tree has ten branches with a strong connection to the central core, determining the ties that unite the constituent elements of the social representation. Each strong branch has a term with a higher degree of connectivity to the core, namely: *hit, leave, approach (n), receive, approach (v), remember, tell, spend, pass, caution, mom*. Such branches constitute the social representation’s peripheral system, which is based on the characteristics of the immediate context, that is, the subjects’ behavior and positions; thus, the branches show the interfaces that guide the nurses’ approach to the victims.

In order to make the presentation of similarity didactic, the terms were organized into four quadrants, namely: right upper quadrant (RUQ), in which the central core emerging from the term *kid* is constituted strongly linked to the terms *approach (n)* and *violence*; the left upper quadrant (LUQ) hosts the 1^st^ periphery including, in the base of the branches, the terms *knock, mother* and *leave (v)*; the left lower quadrant (LLQ) hosts the 2^nd^ periphery including the expressions *beware, pass* and *count*; and the right lower quadrant (RLQ) represents the silent zone with *remember (v), receive* and *approach (v)* linked to *training* and to *feel*.

In the LUQ, the terms *hit, mom* and *leave (v)* were evoked. The word *hit* binds strongly to the term *tell* and this, in turn, branches into *people, professional, turn on, talk, health agent*, and *want*, representative of the PHC professionals. The term *mom* binds strongly to the term *dad* and to term *girl*, while the term *leave* associates with *adolescent* and branches into the term *search for*. Excerpts from the nurses’ statements that represent such branches are as follows:

*Mom came with the child, I also noticed there was no sign of anything and because she spoke even so it seems like it was with a stick she was hitting the child and I guided her, I guided her about the harms it could cause depending on what she was hitting the child with, hitting the child and everything else. (Enf. 18; Level 01; DS 05)*.

*And sometimes even a teenager seeks the service at the clinic, there are few, it’s more when they’re sick even when they get ill, and when the girls come for cytology, it’s more in case of disease, when they’re sick to look for contraceptives, there’s the health agent, we work together. (Enf. 02; Level 01; DS 03)*

*It’s like I talked to you about the health agent, she said: but woman, don’t do anything, don’t do anything”. I said: But woman, I have to go there, I have to investigate, I have to go home”. Then she said: No woman. Then I said: No, don’t worry. I’m not going to go there saying that I know the child is being beaten. (...) she came here, I talked right. And this is the obstacle that we’re sometimes afraid of, do you understand? (Enf. 18; Level 01; DS V 05)*

The term *approach (n)* was evoked from the RUQ, which is linked to the term *violence* and branches, on the one side, under the terms *exist, work, community, unity*; all of these associated with PHC; and, on the other side, in *question* and *service*, as a characteristic of tertiary care, and *nurse* and *reception* of the specialized service. The text segments mentioned by the nurses that refer to this branch are highlighted below:

*And I think that the issue of violence, although it is so present in the daily lives of the communities in which we work, but I think we have been working little, I think we should work more. (Enf. 22; Level 01; DS 04)*.

*This, generally when we arrive, we do the approach, provide the first care according to the prescription, sometimes request the opinion of the social worker, psychology to be able to intensify in other areas. (Enf. 01; Level 03; Sector 03)*.

*It is not always the question that the service is just like that. A screening just watches the caregiver’s history and sees the child’s or adolescent’s conditions, sometime.s (Enf. 07; Level 03; Sector 01)*.

*The nurse’ role here in reception is precisely to carry out this screening to classify what comes from the large demand that comes from the population. (Enf. 09; Level 02; Sector 01)*.

*And the reception time is very fast there, you don’t spend time with the patient. So, there’s no way to do it really, the sector itself is a transit sector. (Enf. 07; Level 03; Sector 01)*.

The LLQ evoked the expressions *caution, pass* and *tell.* The branch from the term *caution* is subdivided into the evocation of the terms *take* linked to *situation* and *case*, which in turn evokes *forward (v), depend, social worker, psychologist*, and *doctor*. And for the other, with the terms: *own, catch, guardianship_council, activate, social_service*, and *call*. The term *pass* connects to *responsible* and *see* and, from the latter, it branches with the terms *story* and *ask*. The central core with the term *tell* is associated with the term *turn* and this divides into *happen* and *problem*. The nurses’ statements related to the terms of such branches are as follows:

*We provided the care and collected the information as soon as we called the psychologist, going to another sector, it is here inside the hospital, I didn’t witness anything.* (Enf. 06; Level 03; Sector 02).*There is the Guardianship Council, right? The social service, the Guardianship Council, so, in this case, when it happens here, we call the social service and the social service will call the Guardianship Council, right? Who will follow the case and see what they do. (Enf. 27; Level 03; Sector 05)*.

*Because the part that is done here at the hospital is care, assistance, when the doctor suspects that the story that the caregiver speaks is not compatible with what the child or adolescent has. (Enf. 07; Level 03; Sector 01)*.

*Generally in all this part, the entire social service takes the lead and the doctors do the reports, the things and they do all the notification. (Enf. 25; Level 03; Sector 04)*.

*Sometimes the child arrives and that’s why I like it when the child speaks. I like to ask the child that sometimes the mother lets the child talk, let her tell what happened. (Enf. 07; Level 03; Sector 01)*.

*In my mind I believe that I should call the NASF, Social Worker and together with her see the place to refer this child or adolescent. (Enf. 04; DS 02)*.

*We always receive guidance to activate the CRAS. To them... Because within the CRAS they activate the Guardianship Council, they see that, they really refer the patient to the destination. But for us to forward, no. (Enf. 08; DS 02)*.

The expressions *remember (v), receive* and *approach (v)* emerge from the RLQ. From the branching of *remember* there is a subdivision into *speak*, on one side and to *perceive* on the other, which connects with *stay, contact, home, patient, team, try* and *get.* The root with the term *receive* branches with the terms *come* and *observe*. The verb *approach* evoked a subdivision with the terms *arrive* and *guide*; from the latter, there are other ramifications, first with *right* and then with the following terms: *find, need, training, feel, family* and *involve*. An isolated weak branch for the term *school* stands out. The excerpts evoked by the nurses that point to the aforementioned terms are found below:

*Sometimes we notice hyperemia or some presence of secretion or even it is a rupture, you can see if registered in the care record, if as part of the physical examination. (Enf. 09; Level 02; Sector 01)*

*But you can see with that clinical look that the bruise that came up there is not a simpler thing, it’s actually an aggression, let’s assume a spot in the eye, there are people who don’t want to say. (Enf. 01; Level 03; Sector 03)*.

*I don’t feel, I don’t feel. So, I feel... I think I have, like, sensitivity, but not the training. So this lacks a lot. (Enf. 22; Level 01; DS 04)*.

*So I think that if there was training, better guidance for us, for the professionals as a whole, it would be much better to handle this situation at the Hospital. (Enf. 15; Level 02; Sector 03)*.

*Last year we had an educational activity at school, in schools about the culture of peace. Where the child was approached together like the schoolchildren’s parents. The topic addressed was this, violence, culture of peace and one of the themes was related to children. (Enf. 20; DS 06)*.

Thus, the following stand out as points of convergence among the nurses at the three health care levels: lack of notifications of situations of violence with children and adolescents; transfer of cases to other professionals; difficulty identifying and confirming violence against children and adolescents; need for training; focus on child care; and difficulty in relationships with the victims’ families.

The underreporting of situations of violence with children and adolescents remain related to the lack of knowledge and approximation with the notification form and confusion between epidemiological notification and notification to the Guardianship Council; added to the mystification of legal and judicial responsibilities, and insecurity to produce the notification considering it as a complaint.

Transferring cases of violence involving children and adolescents to other professionals is associated with the difficulty identifying and confirming the violence linked to the lack of specific training.

Reception is among the points that diverged in the nurses’ performance between the health care levels. In PHC, due to the bonds established with the community, it is based on listening; while in hospital services, reception was based on an action of the conduct-complaint type, with secondary care being based on a Nursing consultation with an initial approach without clinical deepening, with tertiary care assuming a screening position.

**Figure 3:**
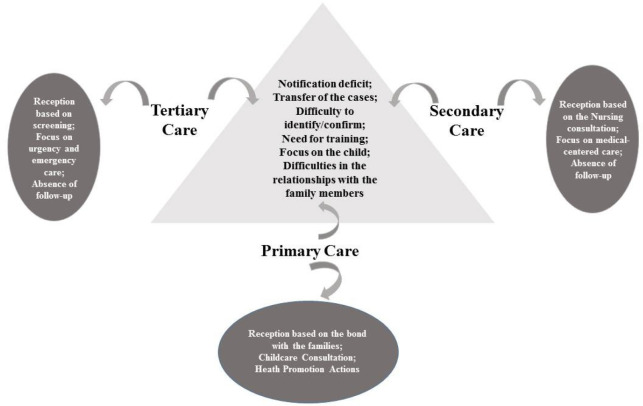
Points of convergence and divergence of the nurses in the approach to children and adolescents in situations of violence at the three health care levels. Campina Grande, Paraíba, Brazil, 2019

Figure 3 condenses the points of convergence and divergence involved in the nurses’ behavior considering the three health care levels, in the structural perspective of the social representations, as it refers to a figure and a meaning^(22)^.

## Discussion

Every social representation is structurally organized around a central core and a peripheral system with the existence of a silent zone; according to the author of the Central Core Theory^(25)^, this premise is constituted from Moscovici’s grand theory itself. The central core is related to collective memory, which generates meaning, consistency and permanence to the representation; thus, it is characterized as stable and resistant to change, being fundamental for the meaning and organization of the representation^(21)^.

In this study, it is pointed out that the central core focuses on the approach to the child who is a victim of violence in the symbolic of the care process performed by nurses, revealing a strongly marked sense in the collective memory of these professionals associated with the child, which can be related to the intrinsic biopsychosocial vulnerability of the child age group, with adolescents in the background.

The central core assumes a meaning- and sense-generating function for the complementary elements (peripheral zone), as it highlights the values and meanings that permeate the behaviors performed by the nurses, presented in the peripheral area of the maximum tree^(25)^; as well as an organizing function that determines the nature of the connections established between the elements of the representation.

In this sense, the elements that characterize the particularities of approaching victims of violence at the different health care levels are explored in the peripheral and silent zone. In the peripheral system, elements common to the nurses who worked at the three health care levels are evidenced; however, the particularities of the nurses’ behavior are also revealed, related to the health service in which they work. Therefore, it is in the peripheral system that the heterogeneity of the representations of the groups is allocated, supporting the contradictions and individual histories in a contextualized manner^(21)^.

In the peripheral system, the nurses’ differentiated practices in approaching children and adolescents who are victims of violence were identified. In primary health care, the approach to these victims has specificities, considering that most of them suffer situations of violence in the domestic environment; knowing and monitoring the families proves to be an essential factor in approaching the cases^(28)^.

This interaction with the family permeates the entire multi-professional team, with emphasis on the Community Health Agents, who, through direct contact with the families, can produce early detection and the establishment of a link with the Basic Health Units that act as a reference and support for the communities^(28-29)^.

A study points out the strength of PHC in confronting violence along two lines: the possibility of horizontal dialogs, lasting relationships and welcoming listening in a process of co-responsibility with the users; and care articulation a health care network, according to the proposed reorganization of the Brazilian health care model, as a protagonist space for network articulations, reflections and interventions^(30)^.

Despite the potential presented by PHC, a number of studies indicate important gaps between the recommendations of health policies aimed at comprehensive care for population groups and assistance to victims of violence in the context of communities and in Basic Health Units^(30-32)^.

The childcare consultations, for being a routine in the nurses’ work process in the BHUs, are shown as the tool for greater access for nurses to aspects of comprehensive child care, as they involve assessment of growth and development, immunization, feeding, specific hygiene care and prevention of accidents, including prevention and identification of situations of violence^(33)^.

Adolescents’ health care has not received assistance in the same proportion, according to the deficit highlighted in the National Adolescents’ Health Survey^(34)^; in it, it was identified that only 48% of the adolescents sought some health service or professional in the last 12 months of the research. Access barriers can hinder this search, such as lack of knowledge about health services or discomfort in sharing health concerns, in addition to the way in which the health teams welcome these adolescents^(34)^.

An important element in producing close relationships between children/adolescents and the Family Health teams comprises the practice of health promotion actions in the spaces of the school, the community and the BHU itself^(35)^. In carrying out these actions, the nurse builds the opportunity to encourage a culture of peace, healthy affective relationships and prevention of violence^(36)^ by identifying risk situations based on the proximity to the children and adolescents.

In the secondary care health service, the care focus is based on the medical-centric approach to violence, centered on the biological body, disregarding subjective, psychological and social aspects^(37)^; although there is a physical and organizational space for the production of care for the victim by the nurse, at this health care level, this practice does not effectively occur as an opportunity for qualified and expanded listening^(38)^.

This point refers to the effectiveness of the Nursing consultation during the reception of children and adolescents who are victims of violence. The need for advances in this scenario is emphasized, from an initial, essential and effective reception, identification of symptoms and signs and notification, also covering the care of injuries and their consequences through records and planning of courses of action in order to achieve a humanized service to the victims, since the Nursing consultation is characterized as one of the main tools for identifying violence against this group^(10)^.

In tertiary care, the focus on the conduct-complaint maintains the hospital-centered view of nurses towards victims of violence, with assistance aimed at remedying the demands of physical urgencies and emergencies. By displaying a behavior focused on the victim’s physical weakness, a number of studies show that emergency services do not recognize, screen or report situations of violence, since the professionals in the emergency rooms are not aware of this phenomenon^(39-40)^.

When it comes to violence against children and adolescents, the nurse should not only focus on evident clinical signs noticeable by inspection, but also on psychosocial indicators from the complete physical examination associated with anamnesis, perception of non-verbal language and physical and emotional needs, in which the professional must establish a trusting conversation to confront the speeches of those responsible and the victims^(41-42)^.

The act of perceiving or recognizing violent situations by nurses applies both to the role of this professional in primary care, by recognizing possible situations of violence in the home environment with children and adolescents, as in the hospital environment, by noticing signs that are not exposed or revealed, especially in the emergency services, a space for greater assistance to child abuse^(9)^.

The identification of child-youth abuse appears as difficulty and inhibits notification of these cases, as shown by a study conducted in Saudi Arabia^(14)^. Identifying violence situations in childhood is complex; it is agreed upon to start the approach of possible violence through the clinical history, as this moment reveals to be powerful to understand the circumstances of the occurrence of the situation of violence; therefore, the nurse must be offer attentive and expanded listening at all times in the face of care in a possible situation of violence with children and adolescents^(43)^.

To produce an approach aimed at the victims’ needs, technical-scientific knowledge is required; the study highlights the notorious fragility of professional training by nurses to approach children and adolescents in situations of violence, which corroborates a recent study^(44)^ involving physicians, nurses and dentists and other studies^(14,45-46)^ that highlight lack of knowledge and the need for training as important barriers to identifying physical abuse with children and adolescents.

This context is a reflection of academic training and insufficient encouragement to train professionals and to define flows and care protocols in the health services, which exerts a direct impact on the feelings expressed by the nurses in the face of cases of violence, as well as on the service performed^(10,16,41)^.

Professional training of nurses to deal with cases of violence with children and adolescents emerged in the silent zone, which corresponds to a subset of meanings, beliefs and cognitions, which, even though they exist, are not expressed in the usual conditions due to the values and norms of the group itself^(25)^. The weakness in the nurse-related training regarding the issue in question is found in the work context; however, in the daily practice, the nurse is socially and institutionally required to provide care, regardless of this subsidy.

Nursing care in conjunction with the multidisciplinary team is characterized as another challenge, marking the difference between sharing care and care transfer permeates the nurses’ actions, who often transfer the cases to the social workers, who establish a link between health care and other protection instances, considering itself as the bridge for resolving cases of violence, and even for identifying suspicious and/or confirmed situations, assuming the vanguard of the cases^(16)^.

This entire context regarding transfer of victims reflects the interruption in the follow-up of cases, which maintains a disarticulation between the health care levels, weakening the referral and counter-referral relationships and favoring revictimization, since the fragmented actions do not cover the complexity of problem. Therefore, the Guardianship Council assumes the concrete role of monitoring children and adolescents without the involvement of the health sector.

In the meantime, the study highlights ways to qualify the nurses’ approach to children and adolescents who are victims of violence in the health services by indicating the weaknesses of each health care level and the specific points of possible interventions from the organization of the work process, instruments of action and training for the nurses’ practice in managing the health services.

As study limitations, the smaller number of nurses who worked in the red room of the emergency health service can be mentioned, as a result of the particularities of the sector’s routine; as well as some health professionals’ difficulty opening to discuss the theme of violence and the praxis itself in dealing with these cases.

The starting point is the premise that there are different ways of knowing and communicating violence to children and adolescents, guided by different objectives of the nurses at the three care levels, ways that are mobile and in which two of them can be defined, in terms of handling or acting on them, which, in turn, are urgent in our societies: the consensual and the scientific^(22)^. In this sense, each nurse, according to the health care level in which they work, generates their own representational universe of violence; this fact, which is situated as a modality of particular knowledge, which has as its function to develop behaviors and communication between individuals in a society that produces meanings^(22)^.

It can be highlighted that the study object permeates the information scaled by the TSR, such as the information, the field of representation and the professional’s attitude towards the complex and polysemic phenomenon of violence against children and adolescents, since that the similarity analysis allows approximating knowledge about the phenomenon using pressure to inference, engagement and information^(22)^.

## Conclusion

The primary, secondary and tertiary health care services from the perspective of the Theory of Social Representations, in the structural perspective of the Central Core Theory, considering the central core, peripheral system and silent zone, showed commonalities in the approach to children and adolescents in situations of violence regarding lack of notification of situations of violence by the nurse; transfer of responsibilities to other professionals in the multidisciplinary team; weak identification of situations of violence with the child-youth group; provision of greater care to the children; difficulty relating to the victims’ families at different times of care; and need for training to approach children and adolescents who are victims of violence.

On the other hand, primary health care presents as particularities the possibility of greater bonding with victims and families, which can favor the approach to cases and the role of nurses; as well as in the development of actions to promote health and encourage a culture of peace. In the hospital health services, the specifics are focused on the medical-centric approach based on the conduct-complaint with low production of bonding and case follow-up.

The gaps in the nurses’ approach to children and adolescents who are victims of violence stand out, evidencing the lack of instruments to subsidize the nurses’ practice in relation to victims of violence. Instrumentalization of the Nursing practice through guidelines, protocols, flowcharts and technical-scientific deepening becomes essential for an effective and precise performance that shall fully meet the victims’ needs.
